# Kinetic and Structural Studies of Aldehyde Oxidoreductase from *Desulfovibrio gigas* Reveal a Dithiolene-Based Chemistry for Enzyme Activation and Inhibition by H_2_O_2_

**DOI:** 10.1371/journal.pone.0083234

**Published:** 2013-12-31

**Authors:** Jacopo Marangon, Hugo D. Correia, Carlos D. Brondino, José J. G. Moura, Maria J. Romão, Pablo J. González, Teresa Santos-Silva

**Affiliations:** 1 REQUIMTE/CQFB, Departamento de Química, Faculdade de Ciências e Tecnologia, Universidade Nova de Lisboa, Caparica, Setubal, Portugal; 2 Departamento de Física, Facultad de Bioquímica y Ciencias Biológicas, Universidad Nacional del Litoral, Santa Fe, Argentina; University of Graz, Austria

## Abstract

Mononuclear Mo-containing enzymes of the xanthine oxidase (XO) family catalyze the oxidative hydroxylation of aldehydes and heterocyclic compounds. The molybdenum active site shows a distorted square-pyramidal geometry in which two ligands, a hydroxyl/water molecule (the catalytic labile site) and a sulfido ligand, have been shown to be essential for catalysis. The XO family member aldehyde oxidoreductase from *Desulfovibrio gigas* (*Dg*AOR) is an exception as presents in its catalytically competent form an equatorial oxo ligand instead of the sulfido ligand. Despite this structural difference, inactive samples of *Dg*AOR can be activated upon incubation with dithionite plus sulfide, a procedure similar to that used for activation of desulfo-XO. The fact that *Dg*AOR does not need a sulfido ligand for catalysis indicates that the process leading to the activation of inactive *Dg*AOR samples is different to that of desulfo-XO. We now report a combined kinetic and X-ray crystallographic study to unveil the enzyme modification responsible for the inactivation and the chemistry that occurs at the Mo site when *Dg*AOR is activated. In contrast to XO, which is activated by resulfuration of the Mo site, *Dg*AOR activation/inactivation is governed by the oxidation state of the dithiolene moiety of the pyranopterin cofactor, which demonstrates the non-innocent behavior of the pyranopterin in enzyme activity. We also showed that *Dg*AOR incubation with dithionite plus sulfide in the presence of dioxygen produces hydrogen peroxide not associated with the enzyme activation. The peroxide molecule coordinates to molybdenum in a η^2^ fashion inhibiting the enzyme activity.

## Introduction

Molybdenum is a transition metal with a high chemical versatility. It is the most abundant transition metal in seawater and an essential constituent of a wide variety of biological systems. Redox-active under physiological conditions, Mo can cycle between the oxidation states IV, V and VI, which make this metal ion an effective transducer between a two-electron and a one-electron redox system [1], [2]. The high chemical flexibility of molybdenum defines its role in enzymatic systems, where it participates catalyzing both oxygen insertion and abstraction in distinct reactions involved in the carbon, nitrogen, and sulfur metabolism [3], [4]. Mo-containing enzymes can be split in two main groups. In the first group the active site comprises a multinuclear heterometallic cluster called FeMoCo which is present in bacterial nitrogenases. The second group comprises enzymes with a mononuclear active site of Mo, which also includes the closely related W-containing enzymes [1], [2]. According to X-ray structural data, primary sequence alignments, and spectroscopic and biochemical features, mononuclear Mo and W enzymes are classified into four broad families, the xanthine oxidase (XO), sulfite oxidase (SO), dimethylsulfoxide reductase (DMSOr), and W-aldehyde oxidoreductase (W-AOR) families [1], [2], [5]–[7]. The XO family members are the best-characterized mononuclear Mo-containing enzymes and, with the exception of CO dehydrogenase and 4-hydroxylbenzoyl-CoA reductase, they catalyze hydroxylation reactions according to
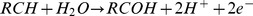



**Figure 1:**
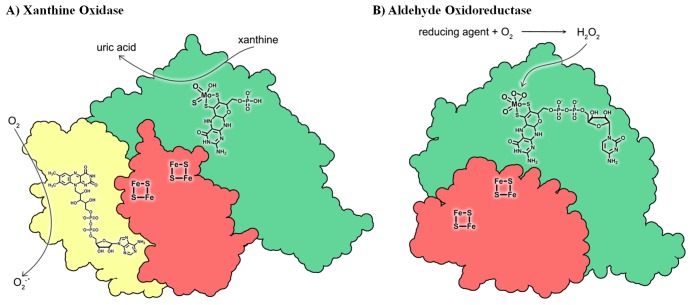
Schematic representation of the domains of bovine milk XO (PDB code: 1FIQ) (A) and *Dg*AOR (B). The Mo, FeS and FAD domains are depicted in green, red and yellow, respectively. The mechanisms of superoxide anion generation by XO as well as the proposal of peroxide generation and binding to the active site of *Dg*AOR are represented. Figure was made using Qutemol [47].

This reaction occurs at the Mo center, and the two reducing equivalents generated are transferred to an external electron acceptor by means of an electron transfer reaction, which in the case of XO is mediated by two [2Fe-2S] clusters and a FAD cofactor. A schematic representation of the domains, cofactor content, and reactions catalyzed by representative Mo-hydroxylases of the XO family is shown in Figure 1A.

**Figure 2:**
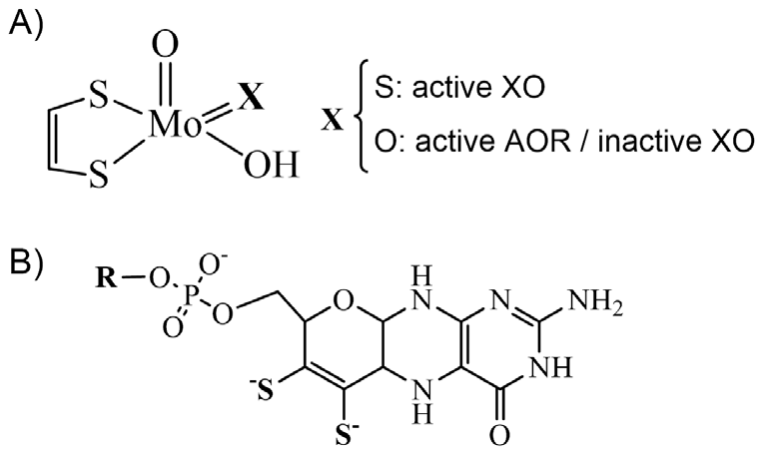
Schematic representations of A) the active site of bovine milk XO and active-*Dg*AOR and b) the structure of the pyranopterin cofactor present in both XO (R = H) and *Dg*AOR (R =  cytidine monophosphate).

Aldehyde oxidoreductase from *Desulfovibrio gigas* (*Dg*AOR, MOP) is a member of the XO family and was the first mononuclear Mo-containing enzyme with reported crystallographic structure [8]. The crystal structure of *Dg*AOR is closely related to that of XO though *Dg*AOR does not harbor the FAD cofactor nor the corresponding domain (Figure 1B) [3], [6], [9], [10]. This enzyme catalyzes the conversion of aldehydes to the respective carboxylic acid at the molybdenum site according to the general reaction described above. The reducing equivalents released from the substrate oxidation flow through the two [2Fe-2S] centers to an external electron acceptor, proposed to be a flavodoxin [11], [12]. The *Dg*AOR physiological role is likely to be related to the generation of reducing equivalents from cytoplasmic aldehydes in order to energize the bacterium cell. However, since a wide spectra of both short- and long-chain aliphatic and aromatic aldehydes are substrates of this enzyme, a detoxifying role should also be considered [13], [14]. Crystallographic studies of as-isolated *Dg*AOR revealed that the molybdenum ion is penta-coordinated in a nearly square-pyramidal geometry. The equatorial ligands of the pyramid are two dithiolene sulfurs of the pyranopterin cytidine dinucleotide (PCD) molecule, one oxo ligand and one hydroxo/water group, whereas a second oxo ligand occupies the axial position (Figure 2) [8], [9]. The coordination around Mo is similar to that of XO, with the only exception being the equatorial oxo ligand, which is a sulfido ligand in XO (Figure 2A) [15]. The equatorial sulfido ligand is essential for XO to catalyze the hydroxylation of hypoxanthine and xanthine, as the XO desulfo-form is inactive [16]. This inactive form of XO, which presents a Mo site identical to that of *Dg*AOR, can be converted to the active form by incubation with dithionite plus sulfide, reaction that substitutes the equatorial oxo group for a sulfido ligand [1], [2].

As we report in this paper, *Dg*AOR can be obtained with different specific activity values depending on the purification batch. Although most purification batches yield fully active enzyme samples, in some cases low specific activity (less than 20% of a fully active sample) or fully inactive *Dg*AOR samples have been obtained. This inactive form of *Dg*AOR (hereafter inactive-*Dg*AOR) can be completely reverted to an active form (hereafter activated-*Dg*AOR) incubating the enzyme in the presence of the strong reducing agents dithionite and sulfide, the method used to activate the desulfo-XO. This fact led to think that both XO and *Dg*AOR share a similar chemistry at the Mo site and hence the X-ray structure of *Dg*AOR lacking the Mo sulfido ligand was associated with the inactive desulfo-form [8], [9]. However, we recently demonstrated that in the as-isolated *Dg*AOR the sulfido ligand is absent and not essential for catalysis [17]. This was concluded through studies that showed that a *Dg*AOR solution prepared from dissolved single-crystals (no sulfido ligand present) and an as-isolated *Dg*AOR sample yielded essentially identical kinetic parameters [17]. Additionally, kinetic studies showed that cyanide, an irreversible XO inhibitor that generates the XO desulfo-form by cyanolysis, is a reversible inhibitor of *Dg*AOR [17].

We report in this paper a combination of kinetic and X-ray crystallography studies to unveil the enzyme modification responsible for enzyme inactivation and the chemistry that occurs at the Mo site when *Dg*AOR is activated in the presence of dithionite and sulfide. Our studies on inactive- and activated-*Dg*AOR reveal the changes experienced by the active site during enzyme inactivation/activation. Additionally, we found that activation of inactive-*Dg*AOR with reducing agents in the presence of dioxygen yields hydrogen peroxide as an undesirable side-product, which led us to study in detail its interaction with the *Dg*AOR active site.

## Materials and Methods

### DgAOR purification and protein quantification

Both active-*Dg*AOR and inactive-*Dg*AOR were purified from *Desulfovibrio gigas* cells cultured under anaerobic conditions with lactate as carbon source and sulfate as final electron acceptor. *D. gigas* cells were treated as described previously [18], [19] to obtain the soluble extract comprising cytoplasmic and periplasmic proteins, and liquid chromatography techniques were used to obtain pure *Dg*AOR up to electrophoretic grade. The soluble extract was loaded into an anionic exchange column containing the DE-52 resin (Whatman), which was previously equilibrated with 5 mM Tris-HCl pH 7.6. The adsorbed proteins were eluted using a linear gradient from 5 to 500 mM Tris-HCl pH 7.6. The fractions containing *Dg*AOR, which were eluted at ca. 250 mM Tris-HCl pH 7.6, were pooled, concentrated by ultrafiltration and then loaded into a column containing hydroxyapatite resin, which was previously equilibrated with 1 mM potassium phosphate pH 8.0. *Dg*AOR eluted with the flow-through while most of contaminants remained adsorbed to the resin. *Dg*AOR fraction was concentrated by ultrafiltration and the final polishing was performed by size exclusion chromatography using a Superdex 200 column equilibrated with 150 mM Tris-HCl pH 7.6. The pure enzyme was concentrated to 150–200 µM and stored in 50 mM Tris-HCl buffer pH 7.6 at 253 K until use.

Active-*Dg*AOR samples were purified from batches showing the highest specific activity values. Inactive-*Dg*AOR samples were purified from batches showing either undetectable or very low AOR activity, following the UV-Vis absorption spectrum signature associated with the two [2Fe-2S] centers of *Dg*AOR. Activated-*Dg*AOR samples were prepared by incubating inactive-*Dg*AOR (150 µM) in the presence of sodium dithionite (30 mM) and sodium sulfide (7 mM) in 50 mM potassium phosphate buffer pH 7.6 under aerobic or argon atmosphere. The excess of dithionite and sulfide was separated by ultrafiltration, the buffer was exchanged to 10 mM Tris-HCl buffer pH 7.6, and *Dg*AOR was concentrated up to 10 mg/mL.

Protein quantification was performed using either the bicinchoninic acid assay (Sigma-Aldrich) or using the enzyme molar extinction coefficient at 462 nm (ε = 24.6 mM^−1^ cm^−1^).

### Kinetic assays

Kinetic studies were performed aerobically at 2981K by measuring the rate of 2,6-dichlorophenol-indophenol (DCPIP) reduction at 600 nm (ε = 21 mM^−1^ cm^−1^) in a 1 cm optical path length cell containing 50 mM tris(hydroxymethyl)aminomethane (Tris-HCl) pH 7.6, 35 µM DCPIP as electron acceptor, 200 µM benzaldehyde as substrate (reaction rate in the V_max_ region), and 450 nM *Dg*AOR. Under these experimental conditions, one enzymatic unit (U) corresponds to 1 µmol of benzaldehyde oxidized per min and the specific activity is U/mg of enzyme.

For the activation experiment monitored through kinetic assays, 150 µM inactive-*Dg*AOR (80% inactive) was incubated in the presence of 30 mM sodium dithionite and 7 mM sodium sulfide in 50 mM potassium phosphate buffer pH 7.6. The incubations were performed at room temperature under argon atmosphere or in the presence of air.

Kinetic assays of *Dg*AOR in the presence of hydrogen peroxide were performed in two ways: a) incubating the enzyme (80 µM) under aerobic conditions with increasing concentrations of hydrogen peroxide up to 5 mM or, b) adding different concentrations of hydrogen peroxide (from 0.5 to 5 mM) to the reaction mixture (see above) with the enzyme under turnover conditions.

### Crystallization, data collection, and refinement

Single crystals of inactive-*Dg*AOR and activated-*Dg*AOR were obtained at 4°C, using the sitting-drop vapor-diffusion method. The precipitating solution contained 30% isopropanol, 0.2 M magnesium chloride in 0.2 M HEPES buffer pH 7.6 as described before [8], [9]. The crystallization drops were prepared adding 4 µL of protein (10 mg/mL) in 10 mM Tris-HCl buffer pH 7.6 to 2 µL of precipitating solution. In both cases, crystals appeared in two weeks and were flash frozen in liquid nitrogen without isopropanol removal.

For soaking experiments, single crystals of active-*Dg*AOR (native) protein were obtained using the same conditions and were then stabilized for at least two days with a harvesting buffer solution (HB1) containing 30% isopropanol, 30% polyethylene glycol 3350, 0.2 M magnesium chloride and 0.2 M HEPES buffer. In order to remove isopropanol from the active site, a second harvesting buffer solution (HB2) containing 30% polyethylene glycol 3350, 0.2 M magnesium chloride and 0.2 M HEPES buffer was slowly added to the drop for two more days. The crystals were then transferred to new drops containing only HB2. After three days of stabilization, the single crystals were soaked for 24 h in a solution of HB2 containing 30 mM sodium dithionite and 7 mM sodium sulfide (dit/S^2−^-soaked crystal), or for 1 hour in HB2 containing 50 µM of hydrogen peroxide (H_2_O_2_-soaked crystal).

Complete data sets were collected at ID14-1, ID14-3, ID23-1 and ID29 of the European Synchrotron Radiation Facility (ESRF, Grenoble, France) at wavelength 0.93 Å. The crystals diffracted up to 1.8-1.5 Å maximum resolution and belong to the P6_1_22 space group with cell constants similar to those of the active-*Dg*AOR (native) protein (PDB code: 1VLB) [8], [9]. Single crystals of active-*Dg*AOR, inactive-*Dg*AOR, and dit/S^2−^-soaked crystal were also measured at a higher wavelength (2.06 Å) in order to enhance the anomalous contribution of sulfur atoms. Data were processed using MOSFLM and SCALA from the CCP4 suite [20]–[22]. Data collection statistics are presented in Table 1. Phases were obtained by molecular replacement using PHASER and the 1.28 Å resolution molecular model of the active-*Dg*AOR (native) protein (PDB code: 1VLB) [9], [23]. The density was improved using DM with 50% solvent content [24]. REFMAC 5.5 was used to perform restrained refinement and COOT was used to inspect the electron density maps and generate water molecules [25], [26]. Geometrical restraints were not used to refine the PCD cofactor. The H_2_O_2_-soaked structure was refined using anisotropic B-factors, during the last stage of refinement. Constructive validation and structure re-refinement was performed using PDB_REDO [27]. Geometrical validation was carried out by several programs such as PROCHECK, MOLPROBITY and STAN [28]–[30]. A summary of the refinement statistics is presented in Table 2. The activated-*Dg*AOR, dit/S^2−^-soaked and H_2_O_2_-soaked structures were deposited in the Protein Data Bank (PDB) database with the codes 4C7Z, 4C7Y and 4C80, respectively.

**Table 1:** Data collection statistics.

Crystal	inactive-*Dg*AOR		activated-*Dg*AOR		dit/S^2−^-soaked		H_2_O_2_-soaked
Beamline	ID14-1	ID29	ID14-1	ID29	ID14-3	ID23-1	ID29
Wavelength (Å)	0.933	2.066	0.933	2.066	0.931	2.060	0.976
Space group	*P*6_1_22	*P*6_1_22	*P*6_1_22	*P*6_1_22	*P*6_1_22	*P*6_1_22	*P*6_1_22
Unit cell (Å)	a,b = 142.2 c = 161.5	a,b = 142.7 c = 161.9	a,b = 143.2 c = 162.3	a,b = 143.1 c = 162.2	a,b = 143.3 c = 162.3	a,b = 142.8 c = 161.7	a,b = 143.1 c = 162.3
Matthews parameter (Å^3^/Da)	2.43	2.45	2.47	2.47	2.47	2.45	2.47
No. observed reflections	683 961 (25 715)	210 477 (20 747)	1 307 618 (52 966)	331 079 (31 720)	715 713 (96 433)	474 177 (62 230)	1 436 348 (213 767)
No. unique reflections	96 794 (4 703)	42 935 (4 458)	141 097 (6 920)	43 108 (4 237)	135 137 (19 580)	53 245 (7 660)	155 276 (22 397)
Resolution limits (Å)	26.73-1.75 (1.78–1.75)	46.70-2.29 (2.38–2.29)	21.26-1.55 (1.58–1.55)	46.84-2.30 (2.38–2.30)	25.68-1.57 (1.65–1.57)	49.99-2.15 (2.27–2.15)	49.58-1.50 (1.58–1.50)
Completeness (%)	99.9 (99.7)	97.2 (97.5)	100.0 (100.0)	98.4 (100.0)	99.5 (99.9)	100.0 (100.0)	99.9 (100.0)
Anom. completeness (%)	-	93.2 (93.2)	-	98.8 (100.0)	-	99.8 (100.0)	-
Redundancy	7.1 (5.5)	4.9 (4.7)	9.3 (7.7)	7.7 (7.5)	5.3 (4.9)	8.9 (8.1)	9.3 (9.5)
Anomalous redundancy	-	2.5 (2.3)	-	4.0 (3.9)	-	4.6 (4.2)	-
Average *I/*σ (I)	16.3 (2.4)	12.5 (2.4)	13.6 (2.7)	22.2 (8.5)	12.5 (3.8)	14.6 (2.8)	20.5 (6.0)
R_merge_ a (%)	7.9 (64.0)	8.4 (54.3)	10.3 (71.9)	6.9 (18.6)	8.9 (35.4)	11.8 (61.5)	6.4 (35.1)

^a^ 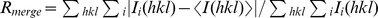, where 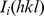 is the integrated intensity of a given reflection and 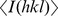
is the mean intensity of multiple corresponding symmetry-related reflections.

Values in parentheses correspond to data in the outermost shell.

**Table 2:** Refinement statistics.

Dataset	inactive-*Dg*AOR	activated-*Dg*AOR	dit/S^2−^-soaked	H_2_O_2_-soaked
PDB Code	-	4C7Z	4C7Y	4C80
Resolution limits (Å)	26.73 - 1.75	21.26 - 1.55	25.68 - 1.57	49.58 - 1.50
Wavelength (Å)	0.933	0.933	0.931	0.976
R-factor (%)	13.66	13.49	13.44	10.33
No. of reflections	91 921	133 832	128 341	147 396
R-free (%)	16.89	15.63	15.79	13.36
No. of reflections (R-free)	4 838	7 072	6 793	7 792
No. of residues	907	907	907	907
No. of atoms	8 422	8 207	8 341	8 137
No. of residues missing	0	0	0	0
Rmsd bond length (Å)	0.010	0.012	0.011	0.010
Rmsd bond angles (deg)	1.682	1.632	1.627	1.628
Average temperature factor (Å^ 2^)				
main chain atoms	15.6	7.5	5.6	13.1
side chain atoms	17.8	9.7	8.1	15.7
water molecules	36.3	27.7	27.0	28.1
Ramachandran plot (%)				
residues in most favored regions	91.6	92.4	91.9	92.4
residues in additionally allowed regions	7.9	7.1	7.7	7.1
residues in generously allowed regions	0.3	0.3	0.1	0.3
residues in disallowed regions	0.3	0.3	0.3	0.3
Overall G-factor	0.07	0.06	0.07	0.09

**Figure 4:**
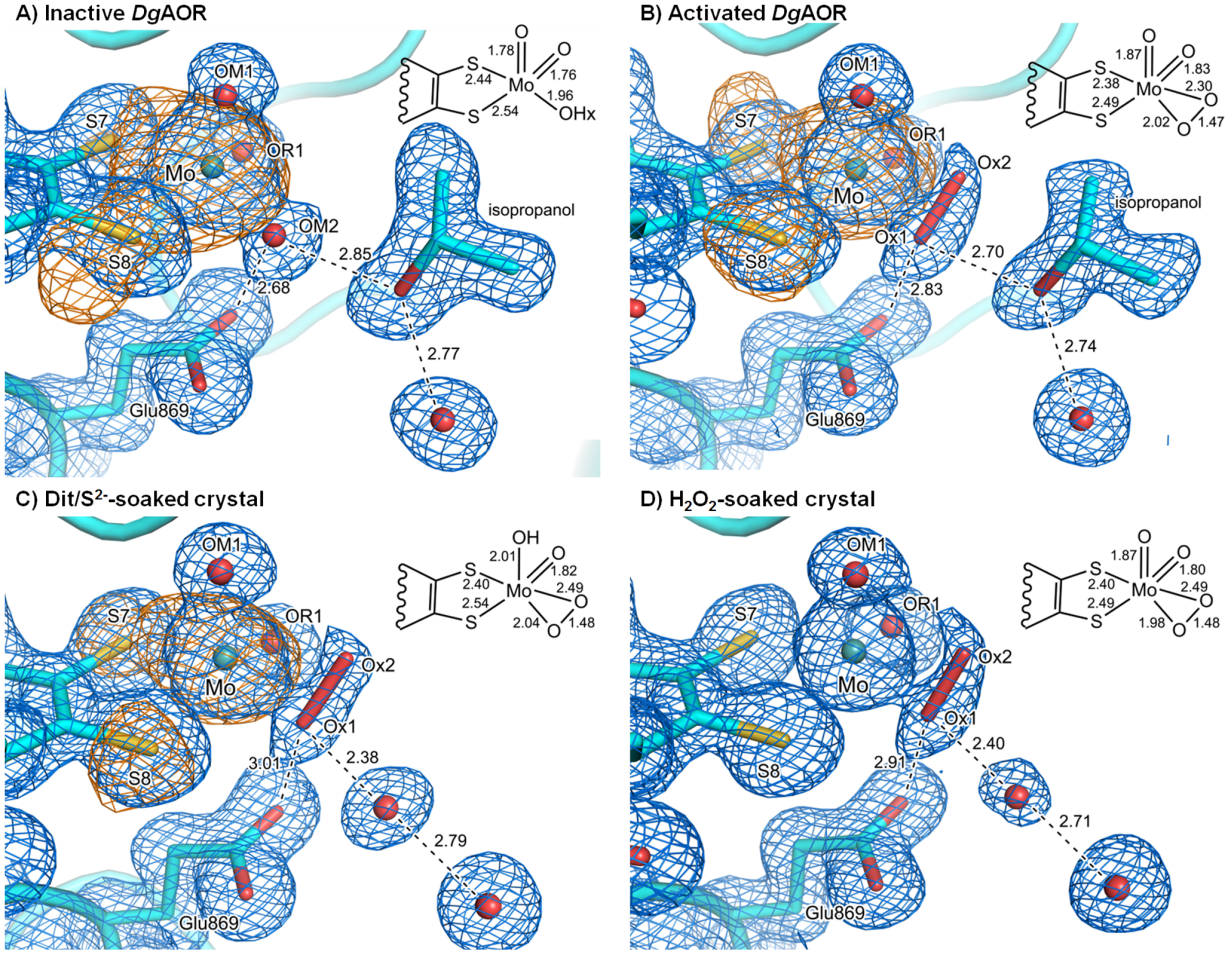
Crystallographic structures of A) inactive-*Dg*AOR, B) activated-*Dg*AOR, C) active-*Dg*AOR crystals soaked with 30 mM sodium dithionite and 7 mM sodium sulfide after isopropanol removal (dit/S^2−^-soaked crystals), and D) active-*Dg*AOR crystals soaked with 50 µM hydrogen peroxide (H_2_O_2_-soaked crystals). Distances are in Å. Atoms color code: Mo in light teal, S in yellow, O in red, C in cyan. The 2mFo–DFc maps (blue mesh) are contoured at 1σ and the anomalous diffraction maps (orange mesh) are contoured at 3σ. The peroxide molecule was modeled in the three structures with occupancies of 1.0 for Ox1 and 0.5 for Ox2.

As expected, the overall structures were highly similar to that of the active-*Dg*AOR (PDB code: 1VLB), with rmsd for all backbone atoms of 0.108 and 0.180 Å for the inactive- and activated-*Dg*AOR, respectively. A careful comparative analysis of both high-resolution X-ray structures revealed that no equatorial sulfido-ligand was observed in any of the structures (Figure 4A and 4B). At this point it was clear that the recovery of AOR activity in activated-*Dg*AOR is not related to the insertion of sulfur atoms either at the Mo-site or elsewhere in the structure. In fact, analysis of the entire protein structure did not reveal differences that could easily explain the enzyme activation and inactivation.

### Spin-trapping and EPR measurements

ROS species produced during the activation procedure were evaluated incubating 75 µM *Dg*AOR or BSA with 3 mM sodium dithionite and 3 mM sodium sulfide. Superoxide generation was performed incubating 2 µM xanthine oxidase with 1 mM xanthine as control. Both reactions were carried out at room temperature and in the presence of air in 100 mM potassium phosphate buffer pH 7.6 containing 100 µM 2-[Bis[2-[bis(carboxymethyl)amino]ethyl]amino]acetic acid (diethylenetriaminepentaacetic acid, DTPA) and 25 mM 5,5-Dimethyl-1-Pyrroline-N-Oxide (DMPO) as spin trap.

X-band CW-EPR spectra were recorded at room temperature (298 K) on a Bruker EMX spectrometer equipped with a rectangular cavity (model ER 4102ST). The microwave power was 10 mW and the 100 kHz modulation amplitude was 1 Gpp. Computer simulations of the spectra were performed using the program WINEPR Simfonia (Bruker Inc.).

## Results and Discussion

### Activation of inactive-DgAOR assessed by kinetic studies

*In vivo*, *Dg*AOR is expressed and performs its task under anaerobic conditions. Yet, it can be purified in the presence of air with its enzymatic activity unaffected. This is supported by the fact that the kinetic parameters obtained in assays performed either under argon atmosphere or in the presence of air are essentially identical (data not shown). Inactive-*Dg*AOR was purified from two batches showing undetectable and very low specific activities (80% inactive). These two samples showed molecular properties identical to those of the active-*Dg*AOR in terms of mass (electrophoretic mobility and mass spectrometry) and content of Mo, Fe and labile S. The kinetic studies were performed with the sample with low specific activity whereas the sample with undetectable activity was reserved for X-ray crystallography studies for a clearer comparison between inactive-*Dg*AOR and activated-*Dg*AOR.

**Figure 3:**
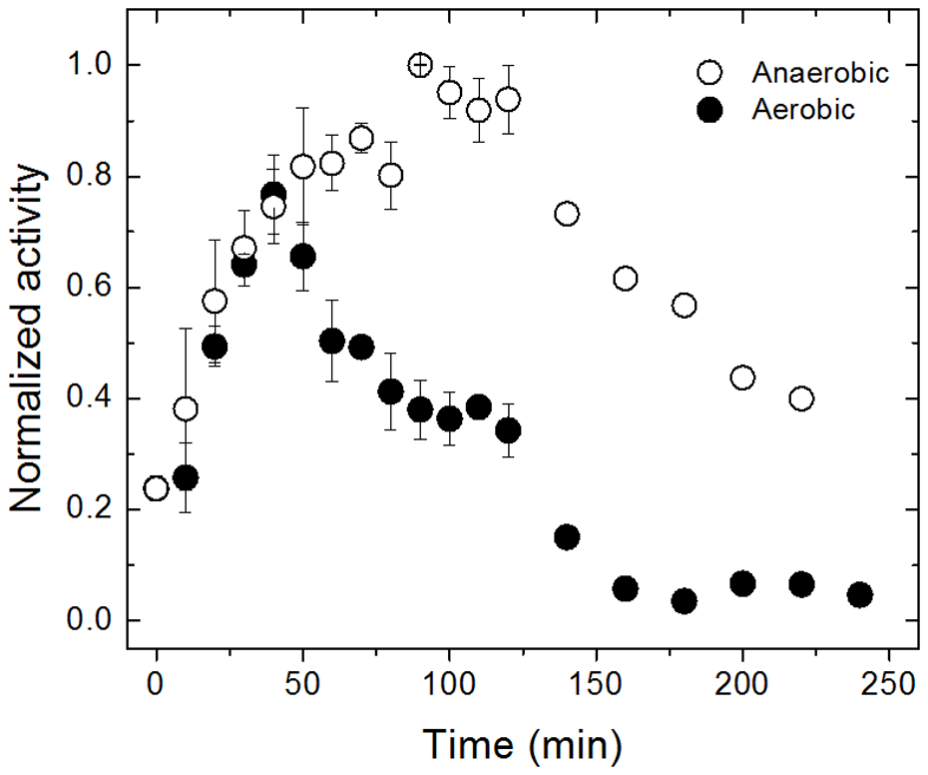
Normalized *Dg*AOR activity vs. incubation time under aerobic (black circles) or anaerobic (empty circles) conditions.

The catalytic competence of inactive-*Dg*AOR was recovered by incubating the enzyme anaerobically for 60 min with sodium dithionite plus sodium sulfide, two strong reducing agents. The specific activity of activated-*Dg*AOR varied from ∼20 to ∼80% compared to active-*Dg*AOR (native enzyme), indicating that the enzyme recovers the activity upon activation. In order to understand the activation process, we monitored changes in specific activity of inactive-*Dg*AOR as a function of time upon activation under aerobic and anaerobic conditions (Figure 3). The different points in Figure 3 were obtained by performing kinetic assays on aliquots taken at different incubation times. It is important to note that for each kinetic assay the reaction was started by addition of the substrate (benzaldehyde). This allowed us to exclude false positives produced by the unspecific reduction of DCPIP by dithionite and/or sulfide contained in each aliquot. This procedure, which allowed us to check the stability of the DCPIP absorbance previous to substrate addition, confirmed that DCPIP reduction was only associated with AOR activity. As shown in Figure 3, the time evolution of the specific activity follows a biphasic behavior under both conditions. In the presence of air, inactive-*Dg*AOR was constantly activated for 40 minutes after which the specific activity decreased, reaching almost complete inactivation after three hours. Under argon atmosphere, the activation phase is longer (120 min) and is followed by an inactivation with moderate slope.

### Molecular basis of *Dg*AOR activation: crystal structures of inactive- and activated-*Dg*AOR

In our previous work we demonstrated that *Dg*AOR does not need the equatorial sulfido ligand to catalyze aldehydes oxidation [17]. The observation that inactive-*Dg*AOR recovers its activity when incubated in the presence of dithionite and sulfide raised the questions whether sulfur is a protagonist in the activation process or the activation is merely triggered by the reducing power of these compounds. In order to solve this issue, we performed X-ray studies on single crystals of inactive- and activated-*Dg*AOR. The inactive-*Dg*AOR was prepared from a batch with undetectable activity and the activation process used to prepare the activated-*Dg*AOR yielded protein with approximately 50% of the specific activity found in active-*Dg*AOR samples.

Single crystals of inactive- and activated-*Dg*AOR were measured using synchrotron radiation at two different wavelengths. The data collected at 0.93 Å have a maximum resolution of 1.75 Å and 1.57 Å for the inactive- and activated-*Dg*AOR crystals, respectively; the data collected at 2.06 Å have a maximum resolution of 2.3 Å for both structures (see table 1 for data collection statistics). The structures, solved by molecular replacement using the native model (PDB code: 1VLB), refined up to final R and R-free values of 13.7% and 16.9% respectively, for the inactive crystals, and 13.5% and 15.6% respectively, for the activated crystals (see Table 2 for refinement statistics).

Anomalous maps were then calculated using data collected at 2.06 Å. At this wavelength, the anomalous contribution of S is higher than at 0.93 Å, enabling a better identification of the presence of sulfur atoms. For the activated-*Dg*AOR structure, a strong anomalous signal was observed for the majority of the S atoms of the structure, either as part of amino acids side chains or in the FeS clusters (not shown). In the active site, anomalous signal was also found for the Mo atom and the two sulfurs of the dithiolene group (S7 and S8) of the pyranopterin cofactor, but not in any other position of the Mo coordination sphere (Figure 4B). This result reinforced the observation that no sulfur atom was inserted in the active site upon treatment of inactive-*Dg*AOR with dithionite plus sulfide.

**Figure 5:**
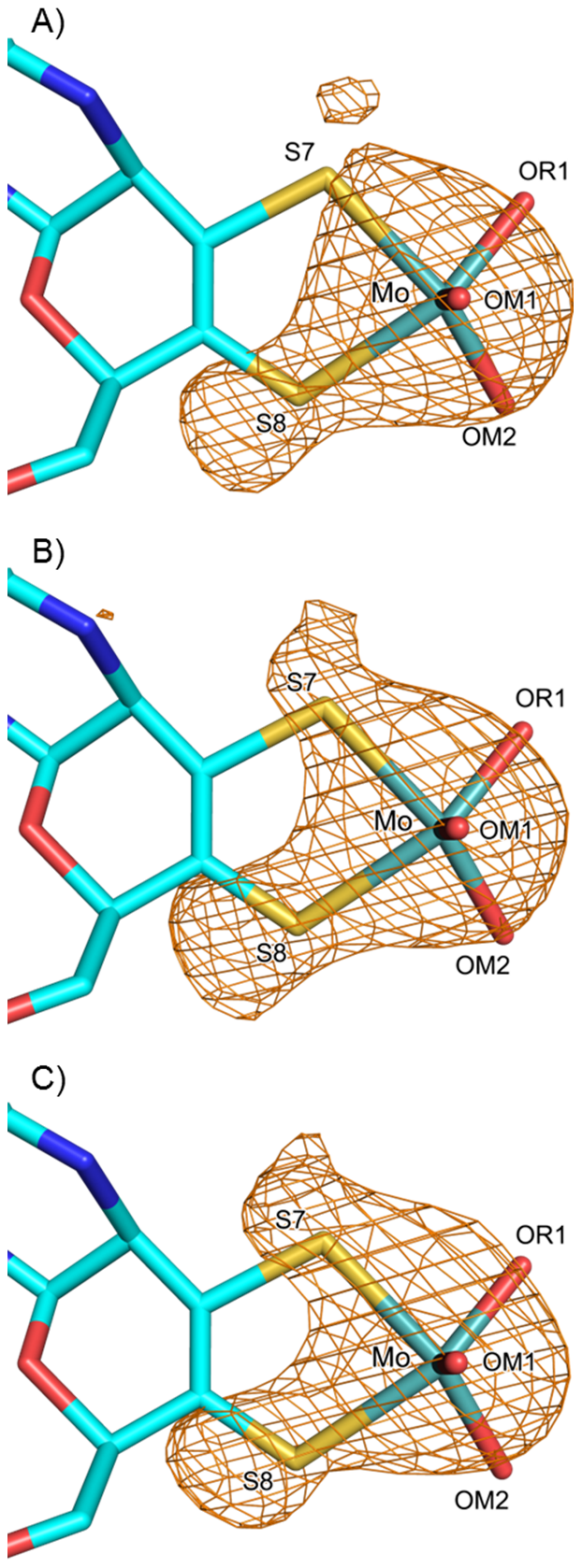
Anomalous difference maps (orange mesh, contoured at 3σ) calculated from data collected at wavelength 2.06 Å for inactive-*Dg*AOR crystal structure using A) 93 images (complete data set), B) the first 73 images, and C) the first 53 images.

The analysis of the anomalous maps for the inactive-*Dg*AOR crystals showed the same anomalous peaks as the activated-*Dg*AOR except for the S7 atom of the dithiolene moiety (Figure 5A). S7 refined well as a sulfur atom with a B-factor similar to those of the neighboring atoms, and no extra peaks in the Fo-Fc maps were observed. The unexpected lack of anomalous electron density at the S7 position is likely produced by site-specific radiation damage that occurred during data collection. In order to test this hypothesis, the same maps were calculated using partial datasets collected at 2.06 Å wavelength. While the map calculated in Figure 5A corresponds to the complete dataset with 93 images (overall 97.2% and anomalous 93.2% completeness), the maps in Figure 5B and 5C were calculated with only the first 73 and 53 images, respectively. Although the overall completeness (oc) and the anomalous completeness (ac) of the data decreases (oc = 88.8% and ac = 80.4% for 73 images and oc = 76.5% and ac = 61.0% for 53 images, respectively), the quality of the maps obtained was very good and showed a gradual appearance of the anomalous signal of S7, indicating that photo-reduction upon X-ray exposure has occurred at this site (Figure 5B and 5C). This means that S7 was in an oxidized state previous to starting the measurements and, from the beginning of data collection, it was gradually photo-reduced as observed from Figures 5C to 5A. The change from an oxidized to a reduced state produces a slight change of the atom position, enough to abolish its anomalous contribution. This phenomenon has been deeply studied for disulfide-bonds in proteins, and several examples reported in the literature showed that photo-reduction induces movement of specific atoms (e.g. sulfurs), causing the disappearance of the anomalous signal and eventually hampering experimental phase determination [31]–[34].

**Figure 6:**
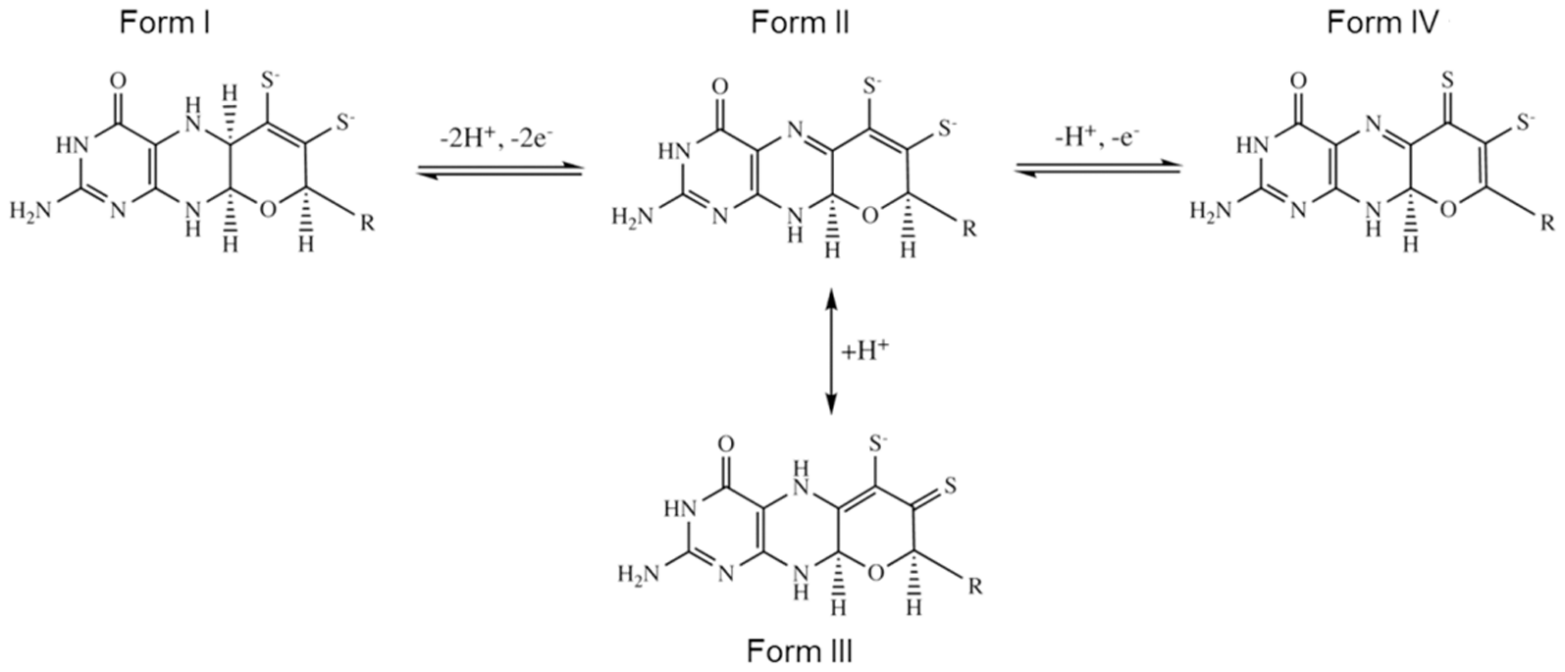
Schematic representations of different pyranopterin forms of the molybdenum cofactor: the reduced tetrahydropyranopterin (Form I); 10,10a-dihydropyranopterin (Form II), a protonated form of the dihydro-pyranopterin possessing a S_7_-thiolene/S_8_-thione moiety (Form III), and a further one-electron oxidation of the dihydro-pyranopterin form is shown in Form IV.

According to these results, the main difference between the inactive- and activated-*Dg*AOR is the oxidation state of S7 of the pyranopterin, which reflects the generally accepted non-innocent behavior of the organic part of the cofactor. The conformation of this cofactor is directly governed by its oxidation state and it has been proposed to have an active role in catalysis by modulating the chemical properties of the Mo atom [35]. The existence of two different oxidation states of the pyranopterin cofactor, namely the tetrahydropyranopterin (form I, fully reduced) and the 10,10a-dihydropyranopterin (form II, 2-electron oxidized), was formerly reported by Matz et al (Figure 6) [36]. According to what was described for Mo-dithiolene model compounds, form II can be further protonated and converted to form III (thione/thiolate) [36]. However, as shown in Figure 5, X-ray exposure induces changes in the oxidation state of S7. This means that, in the inactive-*Dg*AOR, the dithiolene S7 atom presents an oxidation state higher than that of forms I and II (Figure 6), and might be associated to an oxidation state of the cofactor never reported before (form IV in Figure 6). Form IV, like form III, has a net charge of the dithiolene chelate of -1 but it is obtained from form II through a one-electron oxidation step. This type of moiety (thione/thiolate) can coordinate the Mo ion but shift the reduction potentials of the Mo^(n/n−1)^ couples to lower values. This effect translates into enzyme inactivation because the substrates cannot reduce the active site to proceed with the reaction. The structures here presented prompt us to propose that in inactive-*Dg*AOR, the active site might be the form IV of Figure 6, while in active-*Dg*AOR it is in the fully reduced form (form I of Figure 6 and Figure 5A).

In summary, the gradual X-ray photo-reduction seems to revert the inactive oxidized form IV to the original active form of the pyranopterin (forms I and II, Figure 6). The chemical changes experienced by the dithiolene moiety suggest that inactivation of *Dg*AOR is associated with an oxidation process in which the dithiolene S7 is converted from thiolate to thione. When the enzyme is activated, the reducing agents not only reduce the S7 to thiolate but also catalyze the protonation of the C2 atom to yield form II (Figure 6). This confirms that enzyme activation is not associated with sulfur insertion at the Mo site and gives additional support to our previous report that the sulfido ligand is not essential for aldehyde oxidation in *Dg*AOR [17].

### Molecular basis of *Dg*AOR aerobic inactivation during incubation with dithionite plus sulfide

In addition to the oxidation state of S7 from the dithiolene moiety, there is another major difference when comparing the active site of inactive-*Dg*AOR and activated-*Dg*AOR. This difference is related to the labile equatorial ligand trans to S7, which in the inactive-*Dg*AOR (and in active-*Dg*AOR) is a hydroxyl/water molecule, while in activated-*Dg*AOR it is partially replaced by a diatomic molecule (Figure 4B). Based on a detailed analysis of Fo-Fc maps and relative B-factors of the Mo ligands in the activated-*Dg*AOR structure, the diatomic molecule could be modeled with two oxygen atoms (Ox1 and Ox2). Both O atoms coordinate the Mo ion in η^2^ fashion with Mo-O bond lengths of 2.02 and 2.30 Å, respectively (Figure 4B and Table 3). One of the oxygen atoms (Ox1) is hydrogen bonded to Glu_869_ and to the oxygen atom of an isopropanol molecule from the precipitant solution situated close to the Mo cofactor (Figure 4B). This isopropanol molecule is also present in the crystal structure of active-*Dg*AOR structure (PDBcode: 1VLB) in the same position. Both oxygen atoms of the diatomic molecule were refined with different occupancies. Ox1 occupies the position of the labile hydroxo-ligand (OM2) of the active-*Dg*AOR structure and was refined with occupancy of 1, while Ox2 was refined with occupancy of 0.5. This indicates that in the activated-*Dg*AOR crystal structure, two different species have been captured, one identical to the active-*Dg*AOR with the labile hydroxo ligand (OM2), and another where the labile hydroxo ligand has been replaced by the Ox1-Ox2 molecule. The occupancy of 0.5 is in line with the activity recovery of ∼50%. The orientation adopted by the Ox1-Ox2 molecule observed in the structure of Figure 4B resembles that of peroxide in Mo-oxo-peroxo synthetic compounds used to catalyze the oxidation of alcohols to aldehydes, which gave us an initial clue about the nature of the diatomic molecule [37], [38].

**Table 3:** Bond distances between molybdenum and coordinating atoms.

Distance (Å)	active-*Dg*AOR (1VLB)	activated-*Dg*AOR	inactive-*Dg*AOR	dit/S^2−^-soaked	H_2_O_2_-soaked
Mo – S7 (molybdopterin)	2.41	2.38	2.44	2.40	2.40
Mo – S8 (molybdopterin)	2.49	2.49	2.54	2.54	2.49
Mo – OM1 (apical)	1.74	1.87	1.78	2.01	1.87
Mo – OR1 (equatorial)	1.79	1.83	1.76	1.82	1.80
Mo – OM2 (equatorial)	1.99	-	1.96	-	-
Mo – Ox1 (hydrogen peroxide)	-	2.02	-	2.04	1.98
Mo – Ox2 (hydrogen peroxide)	-	2.30	-	2.49	2.49

To evaluate whether the diatomic molecule is related with the activation process, soaking experiments with dithionite plus sulfide were performed on single crystals of active-*Dg*AOR (see Dit/S^2−^-soaked crystal in experimental section for details on sample preparation). The soaking was performed for 24 h to guarantee that the reducing agents diffuse through the entire crystal and in the presence of air to reproduce the experimental conditions used during aerobic enzyme activation. The protein model obtained for the dit/S^2−^-soaked crystals of active-*Dg*AOR was refined and analyzed and, as expected, the overall structure was very similar to that of the active-*Dg*AOR (PDB code: 1VLB) with rmsd of 0.165 Å for all backbone atoms (details on data collection and refinement statistics are given in Table 1 and 2, respectively). Once again, no equatorial sulfido ligand was observed coordinating the Mo, which was confirmed upon inspection of anomalous maps calculated using data collected at the wavelength of 2.06 Å (Table 1, Figure 4C). Similar to that observed for activated-*Dg*AOR, anomalous peaks are present at the Mo and S atoms of the dithiolene moiety but not in any other position of the Mo coordination sphere (Figure 4C). As for activated-*Dg*AOR, a diatomic molecule bound to Mo with 0.5 occupancy was observed. Due to the strong reducing conditions used in this soaking experiment, the oxidation state of the active site in this crystal structure is probably not the same as in the active-*Dg*AOR. During the crystal soaking with dithionite and sulfide, the Mo-site reaches a reduced state, which is denoted by the elongated Mo-OM1 bond distance (Table 3).

The results obtained for the Dit/S^2−^-soaked crystals of active-*Dg*AOR thus confirmed that the generation of the diatomic molecule Ox1-Ox2 is not exclusively associated with the activation of inactive-*Dg*AOR samples. As reported elsewhere, aqueous solutions of concentrated proteins in the presence of dioxygen and strong reducing agents (Krebs solution) generate reactive oxygen species (ROS) among other radical species [39], [40]. This suggests that the diatomic molecule bound to Mo in both activated-*Dg*AOR and Dit/S^2−^-soaked crystal structures might be a ROS originated from the incubation with dithionite plus sulfide under aerobic conditions, being also responsible for the inhibition phase observed in Figure 3.

**Figure 7:**
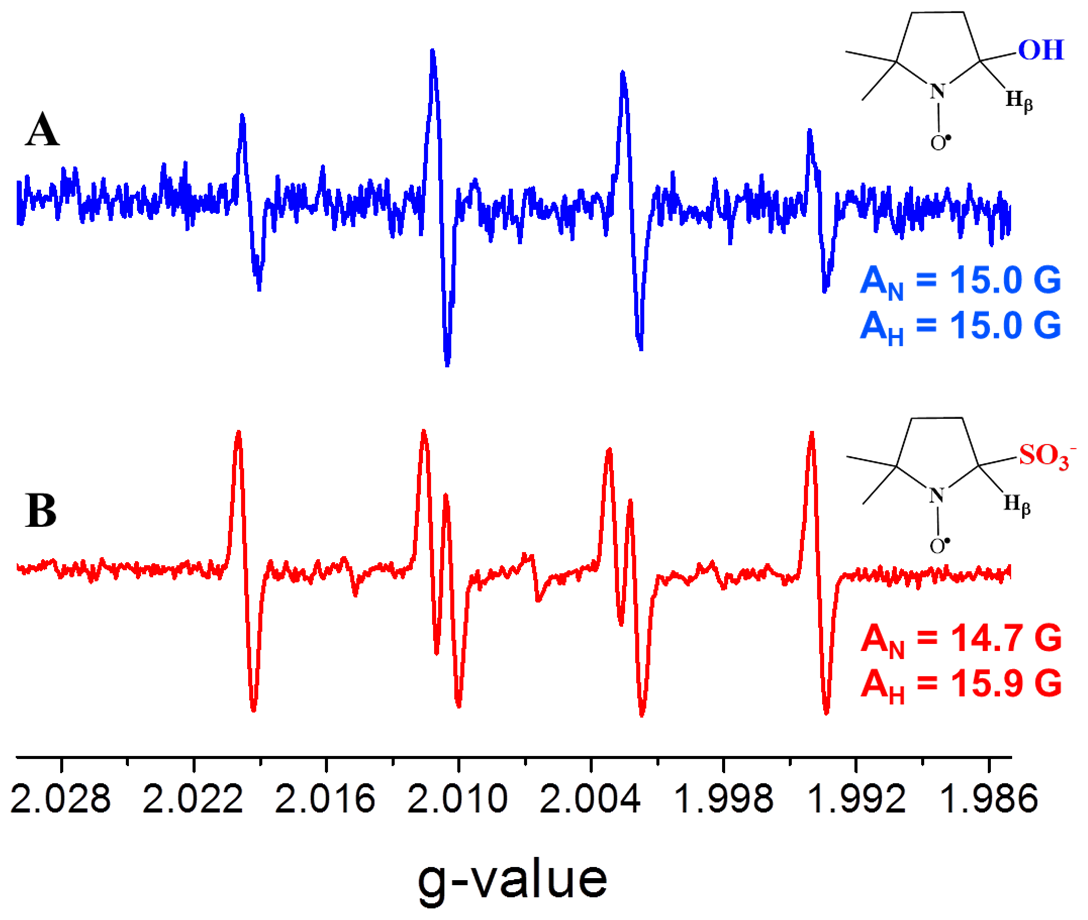
EPR spectra of DMPO-hydroxyl radical (A) and DMPO-sulfite radical (B). EPR parameters (g-values, A_N_ and A_H_) were obtained through computer simulations.

The identity of the diatomic species was assessed by EPR spectroscopy using 5,5′-dimethyl-1-pyrroline-N-oxide (DMPO) as spin trap and xanthine/XO system as control [41], [42]. The latter was used due to the ability of XO to generate superoxide when incubated with xanthine under aerobic conditions in the absence of any other electron acceptor [43]. A scheme showing how dioxygen is reduced to superoxide anion at the FAD-site is shown in Figure 1A, and the EPR signal associated with the radical species formed during superoxide generation by XO is shown in Figure 7A. In contrast to XO, *Dg*AOR does not contain a FAD cofactor and therefore this enzyme is not expected to catalyze dioxygen reduction and generate superoxide (Figure 1B). As expected, the oxidation of benzaldehyde catalyzed by *Dg*AOR under aerobic conditions did not generate superoxide and/or hydroxyl radicals that can be trapped by the DMPO assay (data not shown). However, when concentrated *Dg*AOR was incubated under aerobic conditions in the presence of sodium dithionite and sodium sulfide, an EPR signal typical of a sulfite radical was detected (Figure 7B) [44]. This radical species was also formed when bovine serum albumin was present instead of *Dg*AOR, indicating the unspecific nature of radical species production. Clearly, a sulfite molecule was not the one observed in the Mo-coordination sphere as demonstrated by anomalous maps (Figures 4B and 4C). Therefore, since superoxide was not detected in the spin-trapping experiment, the diatomic molecule coordinated to Mo found in the structure (Figure 4B and 4C) is proposed to be hydrogen peroxide. This hypothesis is in line with previous results on the periplasmic nitrate reductase from *Cupriavidus necator* that showed that peroxides are formed when NapAB crystals are treated with dithionite in the presence of air [40].

The proposed assignment of the diatomic molecule to hydrogen peroxide was supported by crystallographic experiments in which crystals of active-*Dg*AOR were soaked with hydrogen peroxide after isopropanol removal (data collection and refinement statistics are given in Tables 1 and 2, respectively). The model obtained (Figure 4D) was very similar to the dit/S^2−^-soaked crystal structure (Figure 4C) and confirms that the peroxide molecule replaces the labile hydroxyl ligand (OM2) coordinating the Mo ion. Like in the structure of the dit/S^2−^-soaked crystal, hydrogen peroxide was η^2^-bonded to the Mo ion and the analysis of Fo-Fc maps and the B-factors suggested a 50% occupancy for the hydrogen peroxide moiety and the labile hydroxo-ligand OM2.

#### Inactivation of DgAOR by hydrogen peroxide

Kinetic and crystallographic data suggest that the hydrogen peroxide coordinated to Mo is responsible for enzyme inactivation by preventing substrate binding. In order to prove this hypothesis, samples of active-*Dg*AOR (80 µM) were incubated for different times (1, 2, 5, 10 and 20 min) at different H_2_O_2_ concentrations (from 0.05 mM to 5 mM), after which the specific activity of the enzyme was tested (see experimental section). Unexpectedly, H_2_O_2_ concentrations up to 5 mM did not produce inactivation, indicating that active-*Dg*AOR integrity was not affected by high H_2_O_2_ concentrations. However, when H_2_O_2_ was added to active-*Dg*AOR under rapid turnover conditions (H_2_O_2_ addition was performed during the course of the reaction at the beginning of each kinetic assay), the enzyme activity was instantaneously inhibited for H_2_O_2_ concentrations ≥0.5 mM (Figure S1). *Dg*AOR inactivation was also detected in the range 0.1–0.5 mM but with delayed inhibitory effect, while no inhibition was observed below 0.1 mM, which can be explained by the competitive character of the inactivation process. Similar results were obtained when H_2_O_2_ was added previous to substrate addition. It is important to note that reduced DCPIP was not directly oxidized by H_2_O_2_ at the concentrations used in the assays. These evidences suggest that H_2_O_2_ binds irreversibly the Mo ion in a reduced state (during turnover or in the presence of reducing agents). On the other hand, the fact that incubation of active-*Dg*AOR with H_2_O_2_ did not inactivate the enzyme, but that a peroxide molecule was observed in the H_2_O_2_-soaked crystals, suggest that peroxide could bind reversibly to the oxidized Mo ion.

### Molecular basis of DgAOR anaerobic inactivation during incubation with dithionite plus sulfide

The *Dg*AOR inactivation under anaerobic conditions (Figure 3) is less clear than that under aerobic conditions since production of H_2_O_2_ in the absence of dioxygen would be unlikely. Soaking experiments under anaerobic conditions yielded crystals which diffracted very poorly, precluding a definitive explanation of this phenomenon. As demonstrated above, anaerobic incubation of *Dg*AOR with strong reducing agents yields radical species such as the sulfite radical detected by EPR. Then, it is conceivable, although not conclusive, that this radical might be responsible for the anaerobic inactivation of *Dg*AOR, which is not as abrupt as that in the presence of air.

## Conclusions

*Dg*AOR can be purified in two forms, the active-*Dg*AOR (native) and the inactive-*Dg*AOR. Both forms show identical molecular properties and overall crystallographic structures. The inactive-*Dg*AOR form can be activated by incubation with dithionite and sulfide, two strong reducing agents. In contrast to XO, incubation of inactive-*Dg*AOR with dithionite plus sulfide does not incorporate an equatorial sulfido ligand at the Mo site. However, early studies on *Dg*AOR single crystals succeeded in incorporating a sulfido ligand, though it was introduced in the apical position of the Mo coordination sphere [45]. The procedure used for enzyme activation in the present work is different to that used for sulfido incorporation in single crystals, which indicates that the reported “resulfurated” *Dg*AOR form [45] was a particular product of the extreme reducing conditions employed.

The X-ray structures of active-, inactive- and activated-*Dg*AOR here presented strongly suggest that enzyme activation depends on the oxidation state of the Mo-cofactor, and that the dithiolene sulfur atoms and the tricyclic pyranopterin moiety play a key role. The inactive-*Dg*AOR form corresponds to a state in which the S7 atom of the dithiolene moiety is in its oxidized state (thione form, form IV in Figure 6), and the activation implies the reduction of S7 to thiolate (forms I and II in Figure 6). Although the participation of the dithiolene function in modulating the redox properties of Mo-enzymes was previously suggested, this is the first structural evidence regarding the non-innocent behavior of this ligand in enzyme activity.

The procedure used to activate inactive-*Dg*AOR produced radical species and ROS, mainly hydrogen peroxide. The inhibitory effect of H_2_O_2_ was already described in another member of this family of Mo-enzymes, namely in chicken liver XO [46]. However, the interaction of H_2_O_2_ with the Mo-site of XO-related enzymes has not been studied in detail. Our results show that the peroxide molecule binds the Mo in a η^2^ fashion hindering substrate binding. This inhibitory effect is only accomplished when *Dg*AOR is under turnover conditions or in the presence of reducing agents. Considering the involvement of several molybdenum enzymes in oxidative stress (XO and AO), clarification of the hypothetical physiological implication of the Mo-cofactor/ROS interaction is of utmost importance.

## Supporting Information

Figure S1 Normalized *Dg*AOR activity timecourse in a standard assay (black) or when H_2_O_2_ (5 mM) is added during the course of the reaction (red).
(TIF)
