# Supplementary figures and images for: Kinetic and Structural Studies of Aldehyde Oxidoreductase from Desulfovibrio gigas Reveal a Dithiolene-Based Chemistry for Enzyme Activation and Inhibition by H2O2
Source: PLoS One. 2013 Dec 31;8(12):e83234. doi: 10.1371/journal.pone.0083234 (PMC3877041; doi:10.1371/journal.pone.0083234)

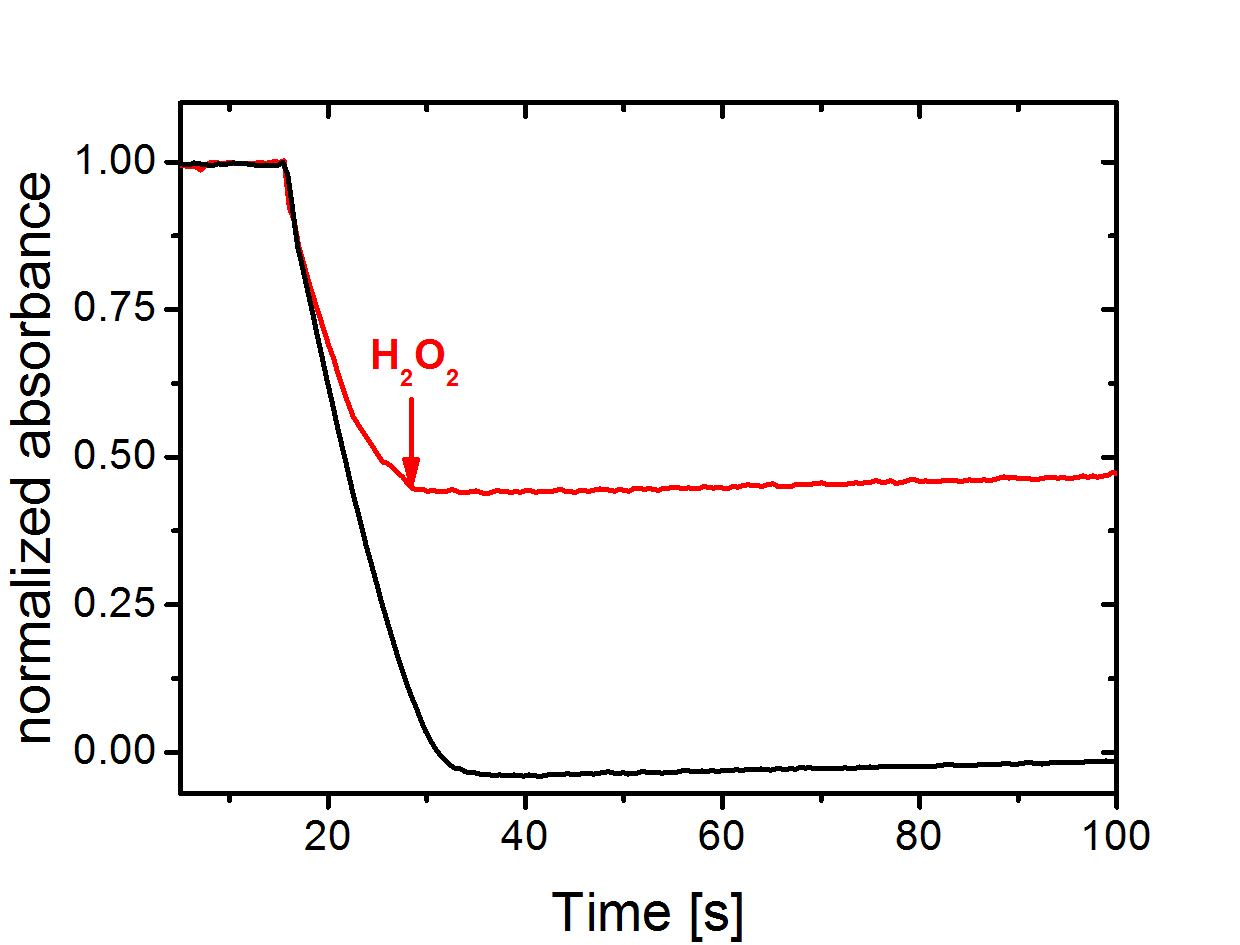

Supplement: Figure S1 — Normalized DgAOR activity timecourse in a standard assay (black) or when H2O2 (5 mM) is added during the course of the reaction (red). (TIF) [file pone.0083234.s001.tif]
